# Amyloid β–dependent neuronal silencing through synaptic decoupling

**DOI:** 10.1073/pnas.2515113122

**Published:** 2025-08-28

**Authors:** Yonghai Zhang, Hsing-Jung Chen-Engerer, Kuan Zhang, Benedikt Zott, Zsuzsanna Varga, Yang Chen, Xiaowei Chen, Hongbo Jia, Bert Sakmann, Israel Nelken, Arthur Konnerth

**Affiliations:** ^a^Institute of Neuroscience, Technical University of Munich, Munich 80802, Germany; ^b^Munich Cluster for Systems Neurology, Technical University of Munich, Munich 80802, Germany; ^c^Brain Research Center and State Key Laboratory of Trauma, Burns, and Combined Injury, Third Military Medical University, Chongqing 400038, China; ^d^Department of Neuroradiology, Technical University of Munich, Munich 81675, Germany; ^e^Institute for Advanced Study, Technical University of Munich, Garching 85748, Germany; ^f^Combinatorial NeuroImaging Core Facility, Leibniz Institute for Neurobiology, Magdeburg 39118, Germany; ^g^Edmond and Lily Safra Center for Brain Sciences, Hebrew University of Jerusalem, Jerusalem 91904, Israel; ^h^Department of Neurobiology, the Silberman Institute of Life Sciences, Hebrew University of Jerusalem, Jerusalem 91904, Israel

**Keywords:** Alzheimer’s disease, neuronal dysfunction, synapse loss, amyloid beta, two-photon imaging

## Abstract

Amyloid β (Aβ)-dependent circuit dysfunction in Alzheimer’s disease (AD) is determined by a puzzling mix of hyperactive and inactive (“silent”) brain neurons. Recent studies identified excessive glutamate accumulation as a key Aβ-dependent determinant of hyperactivity. The cellular mechanisms underlying neuronal silence depend on both Aβ and tau protein pathologies, with an unknown role of Aβ. Here, by using single-cell-initiated rabies virus (RV) tracing in mouse models of β-amyloidosis, we demonstrate that the presynaptic connectivity of silent, but not that of hyperactive, neurons is severely disrupted. Furthermore, silent neurons display a major spine loss and strongly suppressed synaptic activity. Thus, we suggest that synaptic decoupling is an Aβ-dependent cellular mechanism underlying progressive neuronal silencing and a critical factor for the cognitive impairments encountered in AD.

## Significance

A distinct form of dysfunction in Alzheimer’s disease (AD) is a loss of activity in vulnerable neurons, referred to as neuronal “silence”. It has been suggested that neuronal silence is caused by a preceding phase of sustained hyperactivity and represents a prelude to neurodegeneration. While neuronal silence depends on both Aβ and tau protein pathologies, the specific role of Aβ remains unclear. Here, we present direct evidence that structural and functional synaptic decoupling serves as an Aβ-dependent mechanism underlying the severe decline in neuronal activity in AD. This pathological process emerges at an unexpectedly early stage of the disease and progressively intensifies as the disease advances, with severe implications for cognitive performance in the later stages.

Brain atrophy of Alzheimer’s disease (AD) patients, resulting largely from neurodegeneration, is associated with marked impairments in neuronal function. Early stages of the disease with mild cognitive impairments are associated, for example, in the hippocampus, with increased levels of neuronal activity, as indicated by functional MRI (fMRI) recordings (1). Such hyperactivity may worsen the clinical symptoms of AD patients by causing, in addition, epileptiform activity (2). At advanced stages of AD associated with pronounced memory loss and other major cognitive defects, there is a strong disruption of brain connectivity and an overall decline in neuronal activity (1, 3), which coincides with the loss of neurons and brain atrophy (4).

On the cellular level, two-photon Ca^2+^ imaging studies performed in β-amyloidosis mouse models of AD have identified two coexisting forms of neuronal dysfunction consisting of hyperactivity and “silence” of distinct sets of neurons already at early stages of plaque formation (5, 6). The progression of the disease is characterized by the steep increase of both plaque density and the concentration of soluble amyloid β (Aβ), which is paralleled by increasing fractions of hyperactive and silent neurons (7). In recent years, neuronal hyperactivity has been extensively studied by various investigators (5, 6, 8–12). Experimental evidence indicates that the decisive cellular defect is synaptic dysfunction and plasticity through an Aβ-dependent suppression of glutamate reuptake (13, 14). In addition, there is evidence for a role of impaired inhibitory interneuron function (10). By contrast, the mechanisms underlying neuronal silence are less clear and may include excessive synaptic inhibition mediated by hyperactive inhibitory interneurons (15, 16), reduced synaptic excitation as indicated by the loss of spines (17, 18), and/or by the reduction of intrinsic neuronal excitability (19). In addition, there is accumulating evidence for the involvement of soluble tau proteins in neuronal silencing (20, 21). A recent study has identified a specific role of soluble tau proteins in the suppression of CaV2.3 calcium channels, which are essential for neuronal burst firing (22). However, the specific contribution of Aβ-dependent mechanisms for neuronal silence (6, 7) is unknown. Such knowledge is of special interest, given that Aβ accumulation precedes tau accumulation during the progression of AD (23) and may set the stage for the prognostic outcome of the disease.

Not surprisingly, neuronal dysfunction was shown to be associated with circuit dysfunctions (7, 24), ultimately causing cognitive defects such as deficits in learning and memory (24). An important open question is whether circuit malfunction is associated with neuron dysfunction-specific structural changes already at the early stages of the disease. Such an insight could provide a cellular target for interrupting disease progression. A well-suited experimental approach to address this question is single-cell-initiated rabies virus (RV) tracing (25) specifically targeted to functionally defined neurons (26).

## Results

### RV Tracing Initiated in Single Dysfunctional Neurons.

**Fig. 1.**
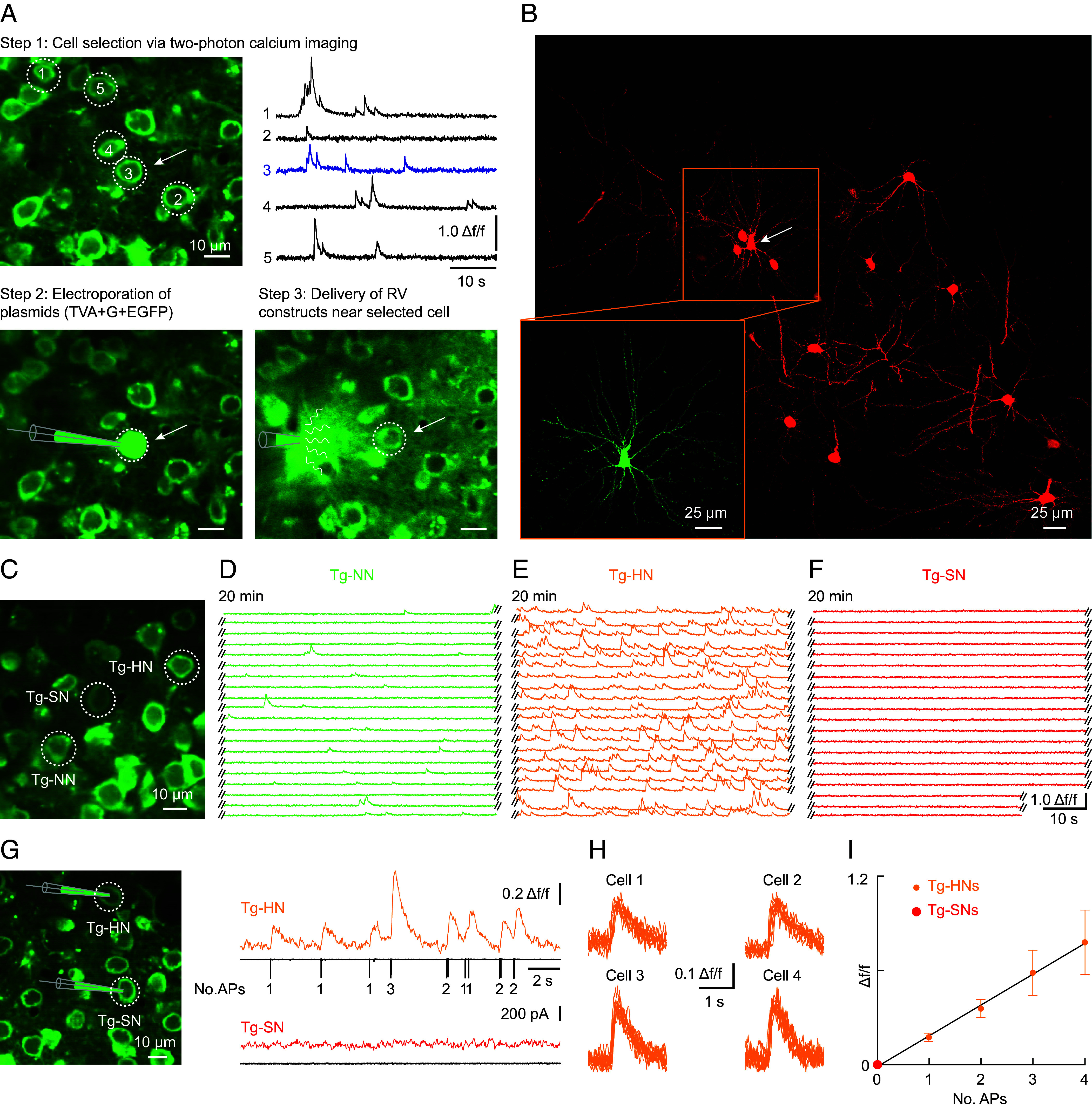
Cell selection and monosynaptic retrograde rabies virus (RV) tracing. (*A*) Single-cell-initiated RV tracing experiment. *Step 1*: In vivo two-photon image of Cal-520 AM-stained L2/3 neurons in the secondary motor cortex (M2) of a WT mouse (*Left*), and spontaneous Ca^2+^ traces (*Right*) recorded in the five neurons circled in the *Left* panel. *Step 2*: Electroporation of the selected “starter” neuron (*arrow*) with a combination of three plasmids (pCMMP-TVA800, pEF1a-G, and pEGFP-N1). *Step 3*: Delivery of EnvA-SADdG-mcherry RV via a patch-pipette near the starter neuron. (*B*) Confocal mCherry fluorescence image of the starter neuron (arrow) and surrounding monosynaptically connected presynaptic neurons. *Inset*: confocal EGFP fluorescence image of the starter neuron. The image was obtained from a PFA-fixed slice prepared 5 d after the initiation of RV tracing depicted in *A*. (*C*) In vivo two-photon image of Cal-520 AM-stained L2/3 neurons in M2 of an APP23xPS45 mouse. (*D*–*F*) Spontaneous Ca^2+^ traces from 20 consecutive 1-min recordings from the corresponding neurons circled in (*C*): Tg-NN—transgenic normal neuron (*D*), Tg-HN—transgenic hyperactive neuron (*E*), and Tg-SN—transgenic silent neuron (*F*). (*G*) In vivo two-photon image of Cal-520 AM-stained L2/3 neurons in M2 of an APP23xPS45 mouse (*Left*). Ca^2+^ recording from the Tg-HN (*orange*) and the Tg-SN (*red*) in combination with the simultaneously recorded action potentials (AP) via cell-attached patch-pipettes (black traces, number of APs as indicated). The recordings from the two neurons were obtained in sequential experimental sessions. (*H*) Single AP-evoked Ca^2+^ transients (superposition of 10 trials) from four hyperactive cells in different APP23xPS45 mice. (*I*) Number of APs vs. somatic Ca^2+^ transient amplitudes (mean ± SD) recorded from four hyperactive neurons in APP23xPS45 mice. Black trace represents the corresponding linear fit. In Tg-SNs, neither Ca^2+^ transients nor APs were detected (red dot at the coordinates of zero, n = 4 neurons). [Scale bar in (*A*), (*C*), and (*G*): 10 µm, in (*B*): 25 µm.]

Pioneering studies by the Callaway lab and others (26, 27) have established the method of single-cell-initiated monosynaptic RV tracing. It is based on delivering the necessary genetic components to individual neurons, mediating fluorescent labeling of monosynaptically connected presynaptic partner neurons. For our studies, we developed a unique approach that combined, in the same experiment, the in vivo identification of dysfunctional single neurons based on two-photon population Ca^2+^ imaging and RV tracing using a modified protocol of a previous study (26). Our recordings were carried out in 6 to 8-mo-old transgenic APP23 × PS45 mice (6) at early stages of Aβ plaque formation (7, 28) and age-matched wild-type (WT) control mice. Fig. 1*A* summarizes the steps involved in a combined two-photon Ca^2+^ imaging and RV tracing experiment in a control WT mouse. The first step of the experiment consisted of bulk-loading of layer 2/3 (L2/3) neurons of the secondary motor cortex (M2) with Cal-520, probably the most sensitive synthetic fluorescent Ca^2+^ indicator currently available (29). Conveniently, Cal-520 neuronal labeling vanishes within a day and, therefore, does not interfere with RV tracing that is performed at a later stage of the experiment. We first selected a neuron based on its firing pattern (Fig. 1 *A*, Step 1). Next, we delivered to this neuron via electroporation several plasmids, including one for expressing avian receptor TVA, one expressing the RV envelope glycoprotein, and one expressing the fluorophore EGFP (Fig. 1 *A*, Step 2). The electroporation was followed by a recovery period of 40 to 60 min, after which we delivered with a second pipette the glycoprotein gene-deleted RV coated with envelope-A (EnvA), the ligand for the TVA receptor, near the electroporated neuron (Fig. 1 *A*, Step 3). Fig. 1*B* illustrates the results of such a single-cell-initiated RV tracing experiment. The confocal image obtained from a post hoc analysis in a fixed slice preparation illustrates in the same optical section the starter cell, a L2/3 pyramidal neuron (green, see inset), and the surrounding presynaptic neurons (red). A low magnification of the section containing the starter neuron is depicted in *SI Appendix*, Fig. S1. Despite its complexity, the approach of RV tracing of functionally pretested individual neurons is very reliable and has a high success rate (>80%).

In the next set of experiments, we established the selection procedure for dysfunctional neurons in transgenic APP23×PS45 mice (Fig. 1 *C*–*F*). By using criteria that were defined in previous studies (6, 28, 30), we identified neurons in transgenic APP23 × PS45 mice (Fig. 1*C*) with normal levels of Ca^2+^ activity (Fig. 1*D*, Tg-NN), hyperactive neurons (Fig. 1*E*, Tg-HN), and silent neurons (Fig. 1*F*, Tg-SN) (*SI Appendix*, Fig. S2). Given the evidence for dysregulation of Ca^2+^ homeostasis in Alzheimer’s models (31) with the risk of somatic Ca^2+^ transients not being faithful reporters of neuronal firing, we combined Ca^2+^ imaging and cell-attached patch-clamp recordings in Tg-HNs and Tg-SNs (Fig. 1*G*). The results demonstrate the accurate detection of single action potential (AP)-evoked Ca^2+^ transients (Fig. 1*H*) and the linearity between Ca^2+^ transient amplitudes and AP-number observed in WT mice (29), without evidence for Ca^2+^ signals in the absence of AP activity in the case of Tg-HNs (Fig. 1*I*).

### Disrupted Synaptic Connectivity of Dysfunctional Neurons.

**Fig. 2.**
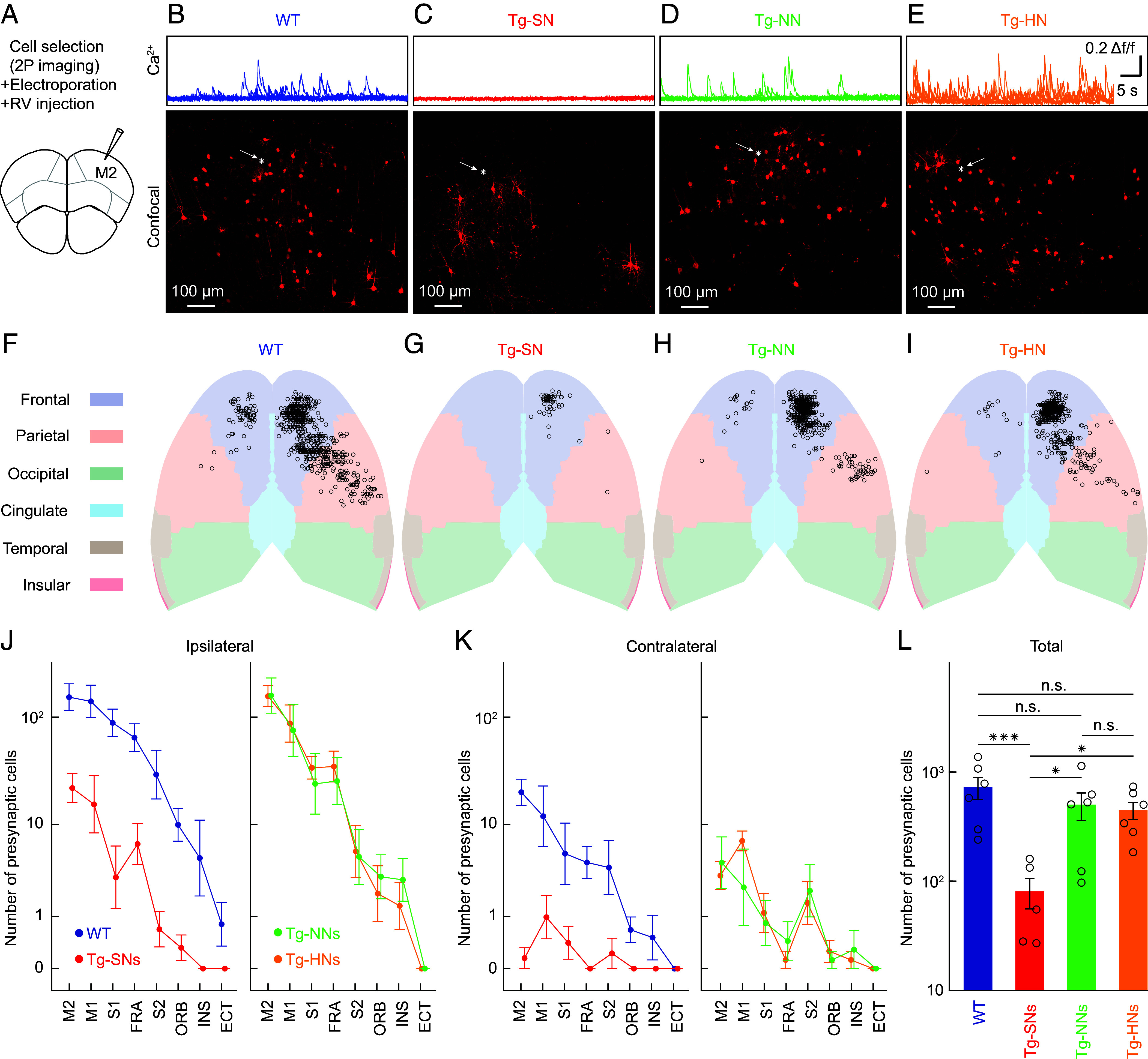
Impaired presynaptic connectivity of dysfunctional neurons in APP23xPS45 mice. (*A*) Schematic of RV tracing initiation in M2. (*B*–*E*) *Top row*: Activity profile of a representative starter neuron in WT, Tg-SN, Tg-NN, and Tg-HN, respectively. Each profile consists of 6 superimposed Ca^2+^ traces reflecting a 1-min recording period. *Bottom row*: Corresponding confocal mCherry fluorescence images with the starter cells (*) indicated by arrows. (*F*–*I*) Distribution maps of presynaptic cortical neurons corresponding to the starter cells in B-E, respectively. (*J*) Ipsilateral cortical distribution of mCherry-labeled presynaptic neurons to single starter neurons (n = 6) in WT mice (*blue*) and Tg-SNs (n = 5) (*red graph*). Similar distribution for Tg-NNs (n = 6, *green*) and Tg-HNs (n = 6, *orange*) (*Right* graph). (*K*) Same as (*J*) for the contralateral cortical distributions for the same starter neurons. (*L*) Total number of cortical presynaptic neurons of the starter neurons in the four different experimental groups. Each circle represents the total number of cortical presynaptic neurons of one starter neuron (WT, n = 6; Tg-SN, n = 5; Tg-NN, n = 6; Tg-HN, n = 6). Abbreviations: M2, secondary motor cortex; M1, primary motor cortex; S1, primary somatosensory cortex; FRA, frontal association cortex; S2, secondary somatosensory cortex; ORB, orbital cortex, INS, insular cortex; ECT, ectorhinal cortex. The data in *J*, *K*, and *L* were plotted as geometric means on a logarithmic axis; error bars depict SEM. ANOVA for log10 of the number of presynaptic neurons as a function of neuron type (WT, Tg-SN, Tg-NN, Tg-HN): F(3, 19) = 8.85, *P* = 0.00070. Post hoc tests were performed using Matlab’s coeftest function. Significant differences between pairs are indicated by the *P*-value. *P < 0.05, **P < 0.01, and ***P < 0.001, n.s. not significant. [Scale bar, 100 µm in (*B*–*E*).]

Using this RV tracing approach, we systematically mapped and compared the presynaptic connectivities in transgenic APP23×PS45 and age-matched WT mice (Fig. 2). The starter cells were always pyramidal neurons in L2/3 of the secondary motor cortex (M2) located 180 to 200 µm below the cortical surface (Fig. 2 *A* and *B*). The comparative analysis of the presynaptic connectivity was performed on day 8 after electroporation. At this time point, the number of transynaptically labeled neurons reaches a plateau, while the starter neuron typically dies from the RV toxicity (26). Notably, in our hands, the number of neurons connected monosynaptically to single L2/3 M2 neurons (721 ± 180, n = 6 experiments) was nearly twice as large as that determined with a similar experimental design for L2/3 mouse visual cortex neurons (26).

The activity status of each starter neuron was tested (Fig. 2 *B*–*E*, *Upper* panels) just before the cellular delivery of the components needed for RV tracing. The four corresponding lower panels in Fig. 2 *B*–*E* show confocal images of cortical sections with mCherry-expressing presynaptic neurons near the injection site. A striking result is the low number of presynaptic neurons connected to the Tg-SN (Fig. 2*C*) as compared to the number of presynaptic neurons in the WT mouse (Fig. 2*B*). The presynaptic connectivities of the Tg-NN (Fig. 2*D*) and the Tg-HN (Fig. 2*E*) appeared to be more similar to that of the WT neurons (Fig. 2*B*). Analogous differences in the distribution of presynaptic connectivity to the starter neurons with different activity profiles were observed also in the other brain regions (*SI Appendix*, Fig. S3). The maps shown in Fig. 2 *F*–*I* illustrate the relative locations of all cortical neurons connecting to the corresponding starter cells described in Fig. 2 *B*–*E*, respectively. Observations from multiple starter neurons with different activity profiles in WT and transgenic animals (*SI Appendix*, Table S1) entirely confirmed the results of the representative neurons shown in Fig. 2 *B*–*I* and emphasized that the most striking insight is the pronounced collapse of presynaptic connectivity to the Tg-SN, which occurs, to a similar extent, in all afferent ipsilateral (Fig. 2*J*) and contralateral (Fig. 2*K*) brain regions (see also *SI Appendix*, Table S1). The remaining neurons connected to Tg-SNs numbered from 33 to 167 neurons (85 ± 28, n = 5 experiments) (Fig. 2*L*). In contrast, while the presynaptic neurons connected to single L2/3 Tg-HNs or Tg-NNs were, in some brain regions, somewhat less abundant, both ipsilaterally and contralaterally, their total numbers were not significantly lower than those connected to control WT neurons (Fig. 2 *J*–*L* and *SI Appendix*, Table S1). Overall, the results demonstrate a highly specific circuit disruption starting at early stages of plaque formation that affects nearly exclusively silent neurons. At the same time, the presynaptic connectivity of Tg-HNs and Tg-NNs appeared to be much less affected.

### Spine Loss and Breakdown of Synaptic Transmission in Silent Neurons.

**Fig. 3.**
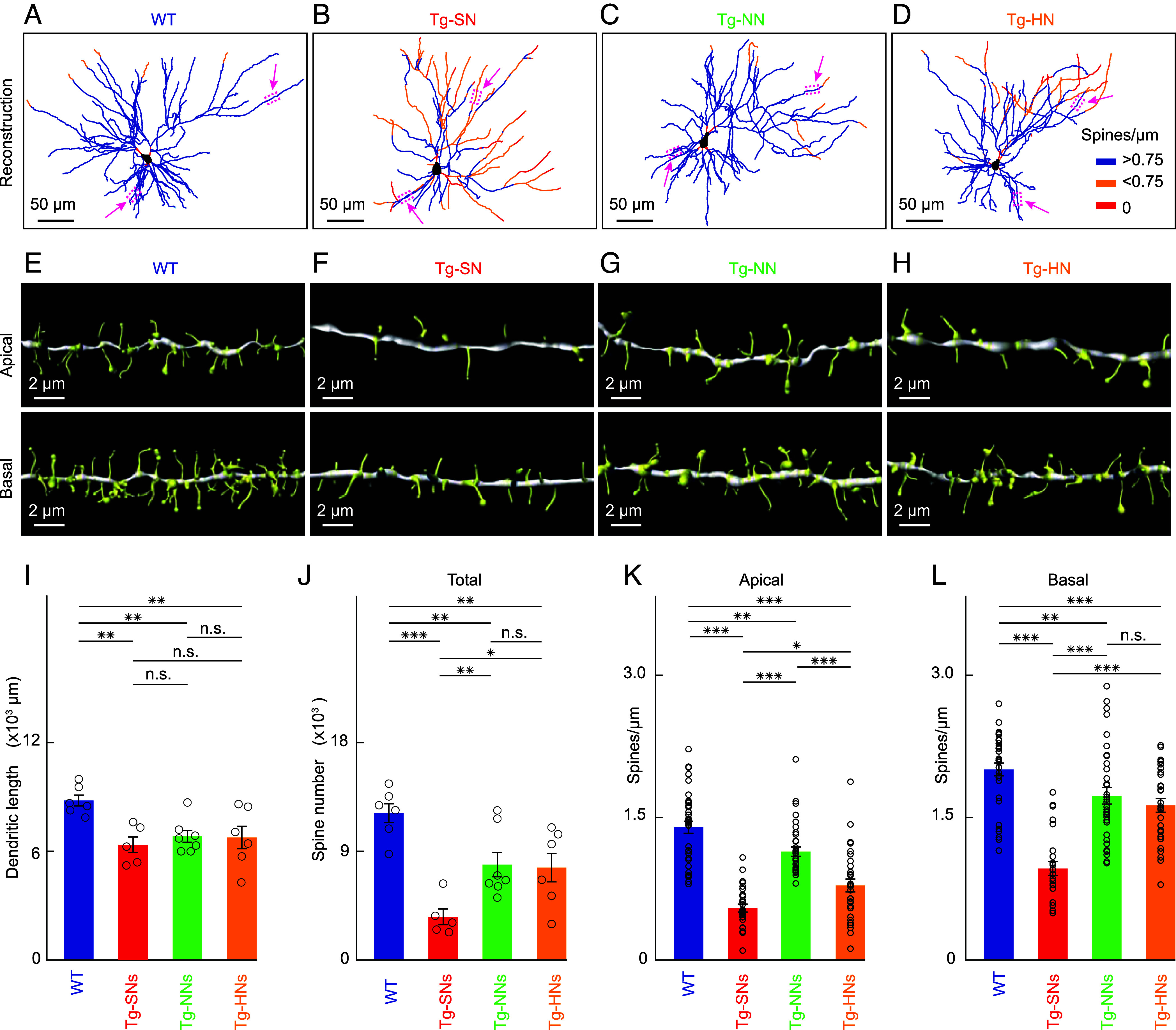
Spine loss and dendritic impairments in dysfunctional neurons. (*A*–*D*) Reconstructions of representative L2/3 M2 neurons in a WT mouse (*A*), and corresponding silent (*B*), normal (*C*), and hyperactive (*D*) neurons in different APP23xPS45 mice. The color code of the respective branches indicates the spine density. (*E*–*H*) 3D reconstructions of the representative apical (*Top*) and basal (*Bottom*) dendritic branches of the neurons in (*A*–*D*). Red arrows in (*A*–*D*) indicate the corresponding branch positions. (*I*) Bar graph summarizing the dendritic lengths of the neurons in different experimental groups. The total dendritic length of individual neurons is indicated by circles (WT, n = 6; Tg-SN, n = 5; Tg-NN, n = 7; Tg-HN, n = 6). (*J*) Bar graph summarizing the total number of spines of the neurons in different experimental groups. Each circle indicates the total number of spines of a single neuron [same neurons as in (*I*)]. (*K*) Spine density in apical dendrites of the neurons in different experimental groups. Each circle corresponds to the spine density of one dendritic segment (WT, n = 36 from six neurons; Tg-SN, n = 30 from five neurons; Tg-NN, n = 37 from seven neurons; Tg-HN, n = 30 from six neurons). (*L*) Same as (K) for basal dendrites (WT, n = 33 from six neurons; Tg-SN, n = 25 from five neurons; Tg-NN, n = 35 from seven neurons; Tg-HN, n = 30 from six neurons). Error bars in (*I*–*L*) depict SEM. ANOVA for the effect of neuron type (WT, Tg-SN, Tg-NN, Tg-HN) on the dendritic length [*I*; F(3, 20) = 5.22, *P* = 0.0080], the total number of spines [*J*; F(3, 20) = 10.2, *P* = 0.0028]. Linear mixed effect models for the effect of neuron type on spine density, with the mouse as a random factor, in apical [*K*; F(3,129) = 18.4, *P* = 5.3*10^−10^] and basal [*L*; F(3,119) = 8.19, *P* = 5.3*10^−5^] dendrites. Post hoc tests were performed using Matlab’s coeftest function. The *P*-value indicates significant differences between pairs. *P < 0.05, **P < 0.01, and ***P < 0.001. n.s. not significant. [Scale bar, 50 µm in (*A*–*D*), 2 µm in (*E*–*H*).]

Morphological changes in central neurons are a prominent feature in the late stages of AD. They consist of a pronounced destruction of all neuronal compartments, especially dendrites and spines in mice (17, 32) and humans (33). To explore whether neuronal dysfunction is associated with specific morphological changes, we adapted our recording protocol and combined functional screening (*SI Appendix*, Fig. S4) with the targeted delivery of the fluorescent morphological marker tdTomato via electroporation of the plasmid to cortical L2/3 neurons in M2 with specific activity profiles (*SI Appendix*, Fig. S2). The fine structure of neurons and their dendritic subcompartments was determined using confocal imaging of serial fixed brain sections (Methods) (Fig. 3 *A*–*H*). The lengths of dendrites, as well as the densities of dendritic spines in apical and basal dendrites, were systematically determined. The dendritic length analysis revealed a significant reduction in total dendritic length across all functional subtypes in APP23×PS45 mice as compared to neurons in WT mice (Fig. 3*I*). There were no significant differences between dendritic lengths of neurons with different types of dysfunction.

By contrast, the analysis of spine numbers and densities revealed remarkable dysfunction-specific changes. In WT neurons (Fig. 3 *A* and *E* and *SI Appendix*, Fig. S5), the spine density was largely similar to that previously observed in L2/3 in somatosensory pyramidal neurons (34). A strikingly different result emerged from the morphological analysis of Tg-SNs (Fig. 3 *B* and *F*), in which the spine number (Fig. 3*J*), the total spine density (*SI Appendix*, Fig. S5), and the spine densities in apical (Fig. 3*K*) and basal (Fig. 3*L*) dendrites were strongly reduced compared to WT neurons. By contrast, Tg-NNs and Tg-HNs displayed to a much lesser extent reductions in total spine number (Fig. 3*J*) as well as spine density in apical (Fig. 3*K*) and basal dendrites (Fig. 3*L*).

**Fig. 4.**
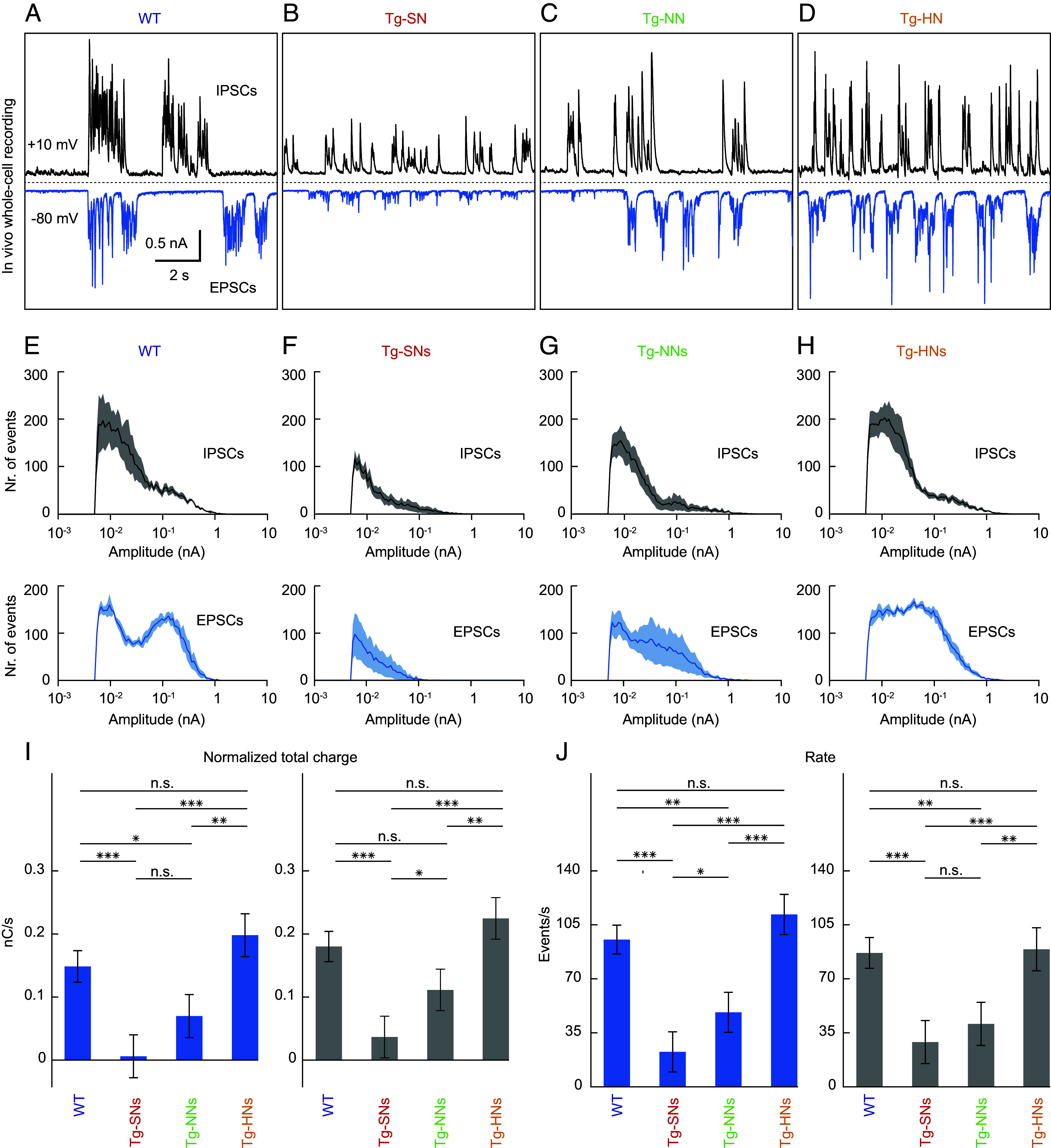
Impaired synaptic activity in dysfunctional neurons. (*A*–*D*) Spontaneous inhibitory postsynaptic (IPSCs) (*Top*) and excitatory postsynaptic currents (EPSCs) (*Bottom*) recorded consecutively in vivo from a representative L2/3 M2 neuron in a WT mouse (*A*), and corresponding recordings from a Tg-SN (*B*), a Tg-NN (*C*), and a Tg-HN (*D*) in different APP23xPS45 mice. (*E*–*H*) (*Top*) Cumulative mean IPSC amplitude distributions obtained in M2 neurons (n = 4) of WT mice (*E*) and Tg-SNs (*F*, n = 5), Tg-NNs (*G*, n = 5), and Tg-HNs (*H*, n = 5) of APP23xPS45 mice. (*Bottom*) Same as the *Top* for the cumulative mean EPSC amplitude distributions obtained in the same neurons. (*I*) Bar graph showing the normalized total charge of the EPSCs (*Left*) and IPSCs (*Right*) obtained in neurons of the different experimental groups [same neurons as in (*E*–*H*)]. (*J*) The same representation as in (*I*) for the rate of the respective synaptic currents in the same neurons. Error bars in (*I*) and (*J*) depict SEM. ANOVA for the effect of neuron type (WT, Tg-SN, Tg-NN, Tg-HN) on excitatory charge (*I*, *Left*): F(3, 15) = 13.7, *P* = 0.00014; neuron type on inhibitory charge (*I*, *Right*): F(3, 15) = 13.4, *P* = 0.00016; neuron type on rate of excitatory events (*J*, *Left*): F(3, 15) = 23.3, *P* = 6.8*10^−6^; neuron type on rate of inhibitory events (*J*, *Right*): F(3, 15) = 11.1, *P* = 0.00042. Post hoc tests were performed using Matlab’s coeftest function. Significant differences between pairs are indicated by the *P*-value. *P < 0.05, **P < 0.01, and ***P < 0.001. n.s. not significant.

Fig. 4 illustrates the results of experiments in which we determined the specific changes in synaptic excitation and inhibition in dysfunctional neurons in transgenic APP23×PS45 mice. We performed in vivo whole-cell voltage-clamp recordings of functionally preselected pyramidal neurons (*SI Appendix*, Figs. S2 and S6). In each case, the selected neuron was first clamped to voltages corresponding to the reversal potential of inhibitory postsynaptic currents (IPSCs) (−80 mV) for the recording of spontaneous excitatory postsynaptic currents (EPSCs) (Fig. 4 *A*, *Lower* trace). After a few minutes of recording, we changed the holding voltage to a value corresponding to the reversal potential of EPSCs (0 to +10 mV) and registered spontaneous IPSCs (Fig. 4 *A*, *Upper* trace). Fig. 4 *B*−*D* displays voltage-clamp traces obtained in the various neuronal subtypes of transgenic animals. The most remarkable results were the dramatically reduced amplitudes of IPSCs and especially EPSCs in Tg-SNs (Fig. 4*B*), while Tg-NNs (Fig. 4*C*) and Tg-HNs (Fig. 4*D*) were much less severely changed.

To quantify possible changes in current amplitude, charge, and rate of the synaptic currents, we applied a parametric deconvolution approach (*SI Appendix*, Fig. S7 and *Materials and Methods*). An in-depth analysis established changes in the synaptic currents in dysfunctional neurons of APP23×PS45 mice. Thus, Fig. 4 *F* and *G* (*Lower* panel), *I*, and *J* validate and extend the dramatic reduction in current amplitude, charge, and rate of both types of synaptic currents in Tg-SNs. The strong attenuation of EPSCs in Tg-SNs is more severe than expected from the changes in spine number (Fig. 3). On the other hand, IPSCs in Tg-SNs were likewise reduced in number, albeit with a lesser reduction of their amplitudes (Fig. 4 *F*, *Upper* panel, *I* and *J*). In line with the moderate morphological changes (Fig. 3), IPSCs in Tg-HNs (Fig. 4*H*) were not significantly different from their counterparts in WT mice (Fig. 4 *I* and *J*). The event rate of EPSCs was even slightly higher than in WT mice (Fig. 4*J*). An unexpected, and only after reflection, a reasonable result was significant reductions in charge (Fig. 4*I*) and rate (Fig. 4*J*) in Tg-NNs for both EPSCs and IPSCs. This result reveals that neurons with “normal” apparent firing properties are by no means necessarily functionally unimpaired but maintain a reasonable E (excitation)/I (inhibition) balance only because of proportional losses in excitation and inhibition (Fig. 4 *I* and *J*). Together, these results demonstrate that the functional decoupling of Tg-SNs was reflected in both excitatory and inhibitory inputs. Moreover, excitatory and inhibitory inputs were impaired to a lesser extent in Tg-NNs and unimpaired in Tg-HNs.

The results presented so far demonstrate a striking correlation between synaptic decoupling and neuronal silence in APP23×PS45 mice. Control experiments in WT mice show that neurons that are “physiologically” silent have an intact synaptic activity (*SI Appendix*, Fig. S8). Such inactive neurons with unharmed synaptic activity were not detected in APP23×PS45 mice. Thus, the breakdown of synaptic activity in Tg-SNs is the consequence of the excessive Aβ accumulation inherent to such mouse models, or perhaps of Aβ-induced tau protein pathologies (22, 35). Indeed, there are APP mutants with low but distinct tau pathology (36, 37). By using immunostaining, we show that in our APP23×PS45 mouse model at the age of 6 to 8 mo, AT8-antibody staining of phosphorylated tau was negative (*SI Appendix*, Fig. S9), suggesting that tau pathology did not contribute to neuronal silencing. Therefore, and in view of earlier reports of neuronal silence in mouse models of β-amyloidosis (6, 7), we conclude that the synaptic decoupling is an Aβ-induced pathology that becomes prominent during the progression of the disease.

## Discussion

Our study reveals a cellular mechanism underlying the gradual loss of action potential activity in vulnerable neurons. The resulting neuronal “silence” was suggested to be the consequence of a preceding phase of sustained hyperactivity (38) and may represent a “prelude” to neurodegeneration, possibly facilitated by the Aβ-dependent elimination of hyperactive synapses (39). Remarkably, while a pronounced decline in brain activity in AD patients is a predominant feature of the later stages of the disease (1, 3), recordings with cellular resolution in mouse models demonstrate that neuronal silence starts already at the early stages of Aβ plaque formation and becomes gradually more pronounced during the progression of the disease (7). Three lines of morphological and physiological evidence identify synapse breakdown as the pathological mechanism of neuronal deactivation. First, in a retrograde monosynaptic RV tracing experiment, we demonstrate a substantial reduction of presynaptic connectivity of Tg-SNs across all ipsi- and contralateral brain areas compared to WT. Second, neuroanatomical analyses of dendrites and spines in dysfunctional neurons of AD mice validate and extend these results by revealing a particularly pronounced loss of dendrites and a decrease in spine density in Tg-SNs. Third, these morphological impairments are paralleled by a massive collapse of afferent synaptic excitatory and a marked reduction of inhibitory synaptic activity in Tg-SNs. Therefore, we conclude that the loss of excitation caused by synaptic decoupling is responsible for the silencing of Tg-SNs, a process that, at later stages, may be aggravated by the accumulation of intracellular tau proteins (20).

In contrast to the marked synaptic decoupling of Tg-SNs, the analysis of Tg-NNs and Tg-HNs revealed only mildly impaired presynaptic connectivity and largely intact dendritic structures. In Tg-HNs, an emerging loss of spines may be overcompensated by glutamate retention in the synaptic cleft at neighboring synaptic inputs (13, 40). Remarkably, the number of active synaptic inputs to Tg-NNs was significantly decreased compared to Tg-HNs (Fig. 4*J*), despite quite similar spine numbers (Fig. 3*J*). Thus, the apparently “normal” neurons, which include most of the neurons in these early stages of the disease, appear unimpaired according to their activity rate, revealing clear changes at the synaptic level. The mismatch between the reduced synaptic activity for a similar number of spines in Tg-NNs compared to Tg-HNs may be explained by an emerging loss of synaptic function preceding the morphological deterioration in a fraction of spines. The unperturbed firing rates of Tg-NNs neurons may result from a parallel reduction in synaptic excitation and inhibition, which prevents a major shift in E/I balance, compensating for the synaptic impairments.

Our findings provide a mechanistic framework for many previous observations of loss of synapses and neuritic dystrophy in AD (18, 33, 41). These changes were suggested to be caused by extracellular Aβ and intracellular accumulation of tau proteins (42, 43). Studies performed in animal models suggested a contribution of Aβ to the observed spine loss (12, 17, 18, 44), but the direct implication for specific forms of neuronal dysfunction remained unclear. Here, we reveal that an initially small population of silent neurons is more severely affected by synapse loss and dysfunction than Tg-NNs and Tg-HNs and is the prime candidate for neuronal degeneration. Moreover, we demonstrate that, at least in early disease stages, impairments of structural connectivity as previously observed by RV tracing experiments in AD mice (45) and structural MRI in patients (46) likely originate primarily from synaptic decoupling of Tg-SNs. This fact is easily overlooked when investigating cell populations. Overall, our findings suggest that synaptic decoupling is an Aβ-dependent cellular mechanism underlying the decline of neuronal activity in AD. This pathological process starts at surprisingly early stages of the disease. It becomes gradually more prominent during the progression of the disease, with devastating consequences for the cognitive performance of the patients at late stages of the disease.

## Materials and Methods

All experimental procedures were performed according to institutional animal welfare guidelines and were approved by the local government.

### Animals.

The experiments made use of 6 to 8-mo-old C57 Bl/6 WT mice of both sexes and age-matched female APP23xPS45 mice. This double-transgenic mouse model expresses both the APP Swedish mutation (670/671) and the G384A mutation in the presenilin 1 (PS1) gene under the Thy-1 promoter (6). Mice were housed in standard mouse cages under a 12-h dark/12-h light cycle at constant temperature and humidity. Food and water were provided ad libitum.

### Implantation of the Head Holder.

Mice were anesthetized with isoflurane (2.0% vol/vol in pure O_2_ for induction, 1.0 to 1.5% for maintenance of the experiment). The body temperature was monitored and kept at 37.0 to 37.5 °C by a heating plate during surgery. A local anesthetic (Lidocaine, 2%) and a short-acting analgesic (Metamizole, 200 mg/kg body weight) were injected subcutaneously. The skull over the motor cortex was surgically exposed, and a custom-built head plate was implanted with dental cement. The exposed bone was covered with a silicone elastomer (Kwik-Sil, WPI). After surgery, a long-acting analgesic was injected subcutaneously (Metacam, 1.5 mg/kg body weight), and the anesthesia was stopped. Mice were then transferred back to the cage for recovery.

### In Vivo Two-Photon Imaging and Characterization of Neurons.

Three days after the implantation of the head plate, each mouse was again anesthetized with isoflurane, as described above. A custom-made plastic chamber was attached to the head plate with cyanoacrylic glue (UHU, Buhl-Baden, Germany). The silicone elastomer above the skull was removed, and a craniotomy of ~1 mm^2^ was made above the secondary motor cortex (AP +2.3, ML 1.6) using a high-speed dental drill (Schick Dental, Schemmerhofen, Germany). The dura was left intact, and the exposed cortex was covered with 1% agarose dissolved in 0.9% saline. After the craniotomy, the mouse was transferred to the recording setup. The recording chamber was perfused with warm (37 °C) artificial cerebrospinal fluid (ACSF) containing 125 mM NaCl, 4.5 mM KCl, 26 mM NaHCO_3_, 1.25 mM NaH_2_PO_4_, 2 mM CaCl_2_, 1 mM MgCl_2_, 20 mM glucose, pH 7.4, bubbled with 95% O_2_ and 5% CO_2_. Multicell bolus loading was performed with the organic Ca^2+^indicator Cal-520 AM as previously described (29, 47). The dye was filled into a glass patch pipette, which was advanced 150 to 200 μm below the cortical surface, and then, the dye was pressure-ejected with 180 to 250 mbar for 1 min. For imaging, the concentration of isoflurane was reduced to 0.6 to 0.8%.

In vivo two-photon Ca^2+^ imaging was performed 1 h after AM dye loading using a custom-built two-photon microscope based on a Ti: Sapphire pulsing laser (model: Chameleon; repetition rate: 80 MHz; pulse width: 140 fs; Coherent, CA) and a scanning unit consisting of a galvanometric mirror and a resonant mirror with a resonance frequency of 12 kHz (Cambridge Technology). The scanner was mounted on an upright microscope (BX51WI, Olympus, Tokyo, Japan) equipped with a water-immersion 40X/0.8 objective (Nikon, Tokyo, Japan). Emitted photons were detected by a hybrid photodetector R7110U-40 (Hamamatsu Photonics, Shizuoka, Japan) together with a DHPCA-100 amplifier (FEMTO Messtechnik GmbH, Berlin, Germany). Full-frame images at 600 × 600 pixels were acquired at 40 Hz or at 450 × 300 pixels at 80 Hz by a custom-written software based on LabVIEW2016 (National Instruments, TX). According to our previous characterization (6), neurons were classified as hyperactive (hyperactive neuron, HN) if they displayed more than four Ca^2+^ transients per minute on average during a 4 to 8 min observation period. Normal neurons were classified as having <4 and >0 transients per minute. To distinguish between normal neurons with low activity levels and silent cells in APP23xPS45 mice, the observation period was extended to 15 to 22 min if the field of view contained at least one cell with zero transients during the initial imaging phase. Only neurons strictly not displaying any Ca^2+^-transients during this extended period were characterized as silent. In WT mice, silent neurons were defined as those having <= 1 transient during a 15 to 20 min observation period.

### Image Analysis.

Two-photon imaging data were analyzed offline as described previously (6, 28). In brief, regions of interest (ROIs) were drawn manually around the somata in the image of the focal plane with custom codes in (National Instruments, TX). Astrocytes were excluded from the analysis based on their specific morphology. The fluorescence changes within each ROI over time were calculated as ΔF/F0 = [F(t) − F0]/F0, where F(t) is the mean fluorescence of all pixels within each ROI at a given time point, and F0 is the mean of the lowest 20% of the ROI intensity values acquired during the entire recording. A fluorescence change was classified as a Ca^2+^ transient if its amplitude was larger than 3 times the SD of the baseline noise. The fluorescence signal was smoothed with an exponentially weighted filter with a time constant of 35 ms (48).

### Targeted Single-Cell Electroporation and RV Injection.

Used plasmids: pCMMP-TVA800 was a gift from Edward Callaway (Addgene plasmid # 15778); pAAV-EF1a-CVS-G-WPRE-pGHpA (Addgene plasmid # 67528) and pAAV-EF1a-tdTomato-WPRE-pGHpA (Addgene plasmid # 67527) were gifts from Botond Roska; pEGFP-N1 was bought from Addgene (Addgene plasmid # 6085-1).

After online analysis of the two-photon imaging data, individual functionally characterized pyramidal neurons were selected and targeted for in vivo electroporation. 8 to 10 MΩ glass pipettes were filled with intracellular solution containing 135 mM K-gluconate, 4 mM KCl, 10 mM HEPES, 4 mM Mg-ATP, 0.3 mM Na_2_-GTP, 10 mM Na-Phosphocreatine, and 1 mM Cal-520 potassium salt. In the RV tracing experiments (Figs. 1*A* and 2), the intracellular solution also contained three plasmids (pCMMP-TVA800, pAAV-EF1a-CVS-G-WPRE-pGHpA, and pEGFP-N1), each at a final concentration of ~200 ng/µl. Alternatively, in the morphological analysis experiments (Fig. 3), only one plasmid pAAV-EF1a-tdTomato-WPRE-pGHpA was added to the intracellular solution at a concentration of ~250 ng/µl.

The targeted single-cell electroporation was performed in the two-photon microscope under visual control using the “shadow-patching” procedure (49). Once the tip of the pipette was in contact with the membrane of the targeted neuron, a train of voltage pulses (−5 V, 7 ms pulse interval, 0.17 ms pulse width, 1 s duration) was given to deliver the plasmids. The electric pulses were generated by the Model 2100 Isolated Pulse Stimulator (A-M system) and were applied to the pipette via the ELC-01MX amplifier (NPI Electronics, Germany). Immediately after the successful electroporation, the neuron appeared bright, due to high levels of intracellular Ca^2+^, and fluorescence levels returned to previous levels after 20 to 40 min, indicating that the cell was not damaged by the procedure. (Fig. 1*A*). In each mouse, only one functionally identified neuron was electroporated.

In the RV tracing experiments, one hour after the electroporation, a second pipette (0.8 to 1 MΩ) containing a mixture of 2 μL of EnvA-SADdG-mcherry RV (titer: 3 × 10^8 ffu/mL) and 0.5 µl fluorescent dye (1 mM OGB-1) was advanced to a site 30 to 50 µm away from the electroporated cell (Fig. 1 *A*3). The viral solution was ejected from the pipette by applying first a high positive pressure (200 to 300 mbar, 2 to 3 s) to unclog the pipette, and then the pressure was lowered to ~80 mbar and maintained for 7 min. After the removal of the pipettes, the craniotomy was sealed by a coverslip and Kwik-cast, and the anesthesia was stopped. Mice were returned to their home cage for recovery.

### In Vivo Single-Cell Electrophysiology.

In vivo electrophysiological recordings were performed using an EPC10 amplifier (USB Quadro Amplifier, HEKA Elektronik, Germany) by the “shadow-patching” procedure (49). For loose-seal cell-attached recordings, the patch pipette (5 to 7 MΩ) solution contained 100 μM OGB-1 dissolved in ACSF. Once the tip of the pipette reached the target cell, a slight suction was applied, and the tip resistance was monitored until a seal of 20 to 50 MΩ was obtained. To analyze the relationship between the number of action potentials (AP) and Ca^2+^ transient amplitudes (Fig. 1 *G*–*I*), Ca^2+^ transients associated with one or high-frequency (>20 Hz) trains of up to four AP were identified and included in the analysis if followed by a quiet interval of at least 100 ms. A linear fit was applied using Matlab.

For in vivo whole-cell voltage-clamp recordings, the patch pipette (3 to 5 MΩ) contained 116 mM Cs-gluconate, 5 mM TEA-Cl, 4 mM Mg-ATP, 0.3 mM GTP, 8 mM phosphocreatine, 10 mM HEPES, 10 mM EGTA, 2 mM CsCl, 2 mM QX-314-Br, and 100 μM OGB-1. The pH value of the internal solution was adjusted to 7.3 with KOH. To record spontaneous EPSCs and IPSCs, the membrane potential was held at −80 mV and +10 mV, respectively, without liquid junction potential adjustment. The series resistance of the pipette was continuously monitored, and neurons were used for recording only if the resistance was <40 MΩ. Electrophysiological data from both cell-attached and whole-cell recordings were filtered at 10 kHz and sampled at 20 kHz using the Patchmaster software (HEKA Elektronik, Germany).

### Analysis of the Whole-cell Voltage-Clamp Recording Data.

All data were processed and analyzed using Matlab (R2020a-R2022b). To avoid ad hoc considerations and arbitrary decisions to the extent possible in the analysis of the postsynaptic current (PSC), we adopted a parametric deconvolution approach to fit the measured current traces as a function of unitary PSC shapes. By studying the local variation of the current traces, we estimated that the amplitude of the measurement noise was about 5 pA. The parameters of this fit were the shape of the unitary PSC. For the unitary events, we used alpha functions u(t) = t^(k−1)^e^(−t/τ)^, having two parameters—the power k and time constant τ. The locations of the unitary events were selected so that their peaks corresponded to the local maxima of the current trace. We thus required the fit to be a sum of unitary events at a predefined set of locations (*SI Appendix*, Fig. S7*A*).

Given the parameters of the unitary events, only the amplitudes of the unitary events remained unspecified. They were estimated by a constrained least-squares procedure, minimizing the summed squared difference between the fit and the measured data while requiring the amplitudes of all unitary events to be larger than 5 pA (the noise level in the measurements). The constraint resulted in setting many of the amplitudes to 0, particularly those related to locations with small local maxima of the current trace. We performed the fit for each neuron and each current type (excitatory and inhibitory) separately. The parameters of the unitary events were different for excitatory and inhibitory currents, with smaller k and longer τ for unitary IPSCs (*SI Appendix*, Fig. S7*B*).

### Histology and Confocal Imaging.

Mice were deeply anesthetized with isoflurane (5%) and transcardially perfused with 0.1 M PBS, followed by 4% paraformaldehyde (PFA) in PBS two or seven days after electroporation for morphological analysis or RV tracing experiments, respectively. The brains were removed and postfixed in PFA overnight at 4 °C. The entire cerebral cortex was cut into coronal sections of 80 μm using a vibratome (Leica VT1000S, Leica Microsystems, Nussloch GmbH, Germany). Images were acquired on an inverted confocal laser-scanning microscope (FV3000, Olympus, Tokyo, Japan). For the RV tracing, slices were imaged with a 4×/0.16 NA dry UPLSAPO objective (Olympus, Tokyo, Japan) or a 20×/0.85 NA oil immersion UPLSAPO objective (Olympus, Tokyo, Japan) with a Z-spacing of 1 µm and a pixel resolution of 310 nm pixel^−1^. For Fig. 1*B* we imaged the slice with a 60×/1.42 NA oil immersion PLAPON60X objective (Olympus, Tokyo, Japan) with a Z-spacing of 1 µm and a pixel resolution of 104 nm pixel^−1^. For the morphological analysis experiment, slices were imaged with the 60×/1.42 NA oil immersion PLAPON60X objective (Olympus, Tokyo, Japan) with a Z-spacing of 0.5 µm and a pixel resolution of 52 nm pixel^−1^.

### Immunohistochemistry.

For the detection of phosphorylated tau using AT8 immunohistochemistry (50), mice were anesthetized and transcardially perfused as described above. In a set of experiments, the effect of hypothermia was examined in mice kept at room temperature for 1 h before perfusion (51). The brains were embedded in 4% agarose, and 40 to 50 µm-thick coronal sections were cut with a Leica VT1000S vibratome. Slices were washed in TBS (50 mM Tris, pH 7.5) for 4 × 5 min and incubated in blocking solution (5% normal goat serum and 0.3% Triton X-100 in TBS) for 1 h. After washing again (3 × 5 min in TBS), slices were incubated in the anti-tau primary antibody (clone AT8, MN1020, ThermoFisher Scientific, RRID AB_223647) diluted 1:1,000 in the blocking solution for 1 night. The next day, slices were washed in TBS (2 × 5 min), 0.1% TritonX-100 in TBS (2 × 5 min), and in TBS again (2 × 5 min), and incubated in Alexa Fluor-coupled secondary antibody diluted to 4 µg/ml in blocking solution (A-21240, Invitrogen-ThermoFisher Scientific, RRID AB_2535809) for 90 min. After washing (TBS 2 × 5 min, 0.1% TrintonX-100 in TBS 2 × 5 min, TBS 3× 5 min), slices were mounted in DAPI-containing Vectashield Plus mounting medium (H-2000, Vector Laboratories) and imaged on the following day. Images were acquired on a Fluoview FV3000 (Olympus-Evident) confocal scanning microscope using the UPLSAPO20XO/0.85 objective. Image resolution was set to 155 nm pixel^−1^, and the z-step size was 2 µm.

### Confocal Image Analysis.

Confocal images were processed with ImageJ (https://imagej.net/ij/index.html) software. In the RV tracing experiments, we first determined the distance of each brain section from Bregma based on coronal maps of the mouse reference atlas (52). Then, the presynaptic mCherry-expressing neurons contained in the section were labeled manually and their coordinates were documented and assigned to a brain region based on a mouse reference atlas (52). Individual neurons were projected onto a simplified two-dimensional representation of the cortical surface (53, 54) according to their recorded coordinates (Fig. 2 *F*−*I*). For the morphological analysis experiments, neuronal structures were traced manually using the Filament Editor of the Amira software (Thermo Fisher Scientific, MA). Spine numbers were counted manually, and their coordinates were documented in the z-stack images using ImageJ software. The coordinates of the spine were loaded into a custom-written code in LabVIEW 2016 (National Instruments, TX) to calculate the spine density and draw the density map. The 3D morphological structure of dendrites was reconstructed using Imaris filament tracer v. 8.4.2 (Bitplane, MA).

### Statistics.

All statistical analyses were performed using Matlab (R2020a-R2022b). The data from the RV experiments were logarithmically transformed to reduce the right skew of count distributions. To avoid the singularity at 0, 0.5 was added to all numbers before the transformation. For the morphological analysis and electrophysiological experiments, the statistical analysis was performed based on the original data.

The fitlm function in Matlab was used to fit linear models for the effect of neuron type (WT, Tg-SN, Tg-NN, Tg-HN) on the number of presynaptic neurons, the dendritic length, the total number of spines, and the spine density in apical and basal dendrites, and the synaptic inputs to single neurons, followed by corresponding ANOVA test. Post hoc tests were performed using Matlab’s coefTest function. In all analyses, *P* < 0.05 was considered to be statistically significant. Data are expressed as means, and the error bars depict SEM unless specified otherwise in the legends.

## Supplementary Material

Appendix 01 (PDF)

## Data Availability

All supporting data and custom analysis scripts have been deposited in Zenodo (https://doi.org/10.5281/zenodo.16837260) (55). All other data are included in the article and/or *SI Appendix*.
