# Supplementary material for: Amyloid β–dependent neuronal silencing through synaptic decoupling
Source: Proc Natl Acad Sci U S A. 2025 Aug 28;122(35):e2515113122. doi: 10.1073/pnas.2515113122 (PMC12415221; doi:10.1073/pnas.2515113122)
Supplement: Supplementary file 1 — Appendix 01 (PDF) [file pnas.2515113122.sapp.pdf]

PNAS

Supporting Information for

Amyloid β-dependent neuronal silencing through synaptic decoupling

Yonghai Zhang[1,2], Hsing-Jung Chen-Engerer[1,2], Kuan Zhang[1,2,3], Benedikt Zott[1,2,4,5], Zsuzsanna Varga[1,2], Yang Chen[1,2], Xiaowei Chen[3], Hongbo Jia[1,2,6], Bert Sakmann[1*], Israel Nelken[1,2,7], Arthur Konnerth[1,2*]

*Corresponding authors: Bert Sakmann (bsakmann@neuro.mpg.de) and Arthur Konnerth (arthur.konnerth@tum.de)

This PDF file includes:

Figures S1 to S9
Tables S1
SI References

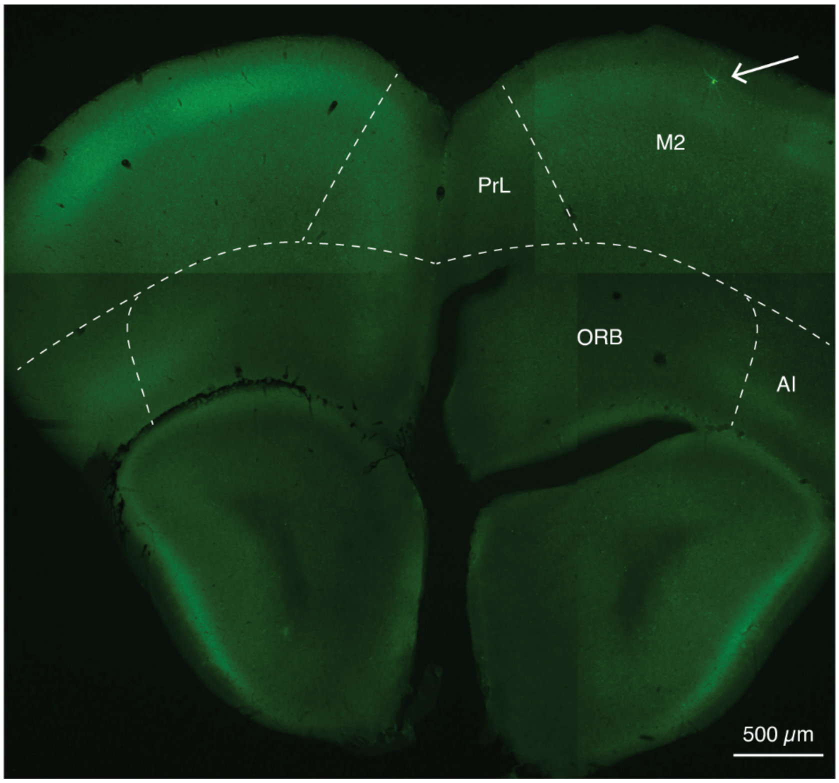

Fig.S1. Location of the starter neuron in Fig. 1B. Stitched confocal image of a coronal brain section containing the EGFP-expressing starter neuron (white arrow) in layer 2/3 of the secondary motor cortex (M2). The borders of the cortical regions (dotted white lines) were assigned according to the mouse brain atlas (1). Abbreviations: M2, secondary motor cortex; ORB, orbital cortex; PrL, prelimbic cortex. AI, agranular insular cortex. Scale bar: 500 μm.

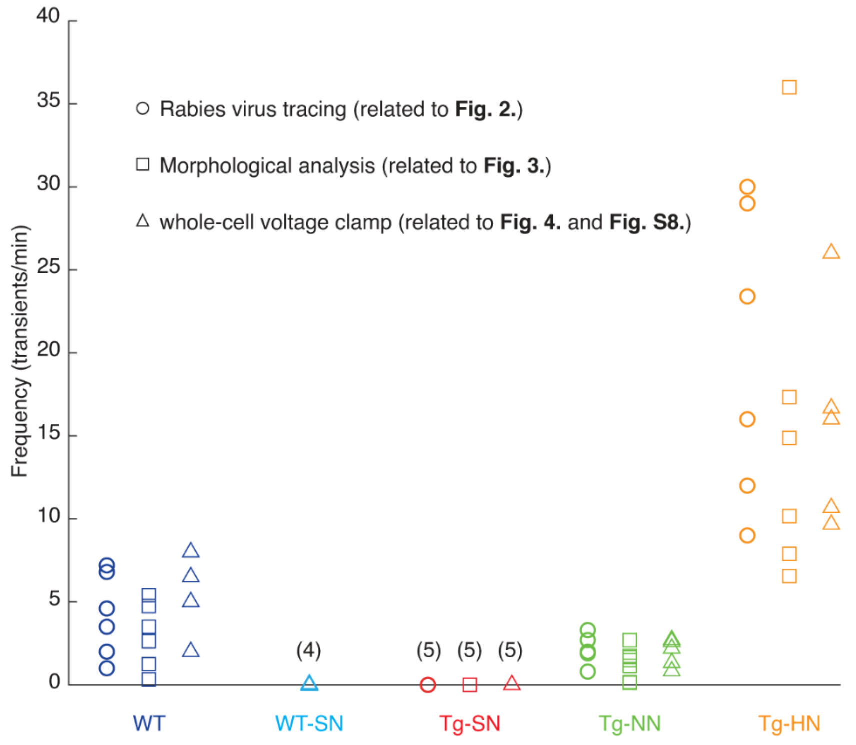

Fig.S2. Frequency of Ca^{2+} transients of the sampled neurons across all experiments. Average frequency (Ca^{2+}-transients/min) of the neurons used in rabies virus tracing (○), morphological analysis (□), and in vivo whole-cell voltage-clamp (Δ) experiments. Each symbol corresponds to an individual neuron. For silent neurons, the frequency was below 0.1 in the WT-SN group (n = 4) and was zero in all instances in the Tg-SN group (n = 5 for each experiment, respectively), and in consequence, the symbols are superimposed.

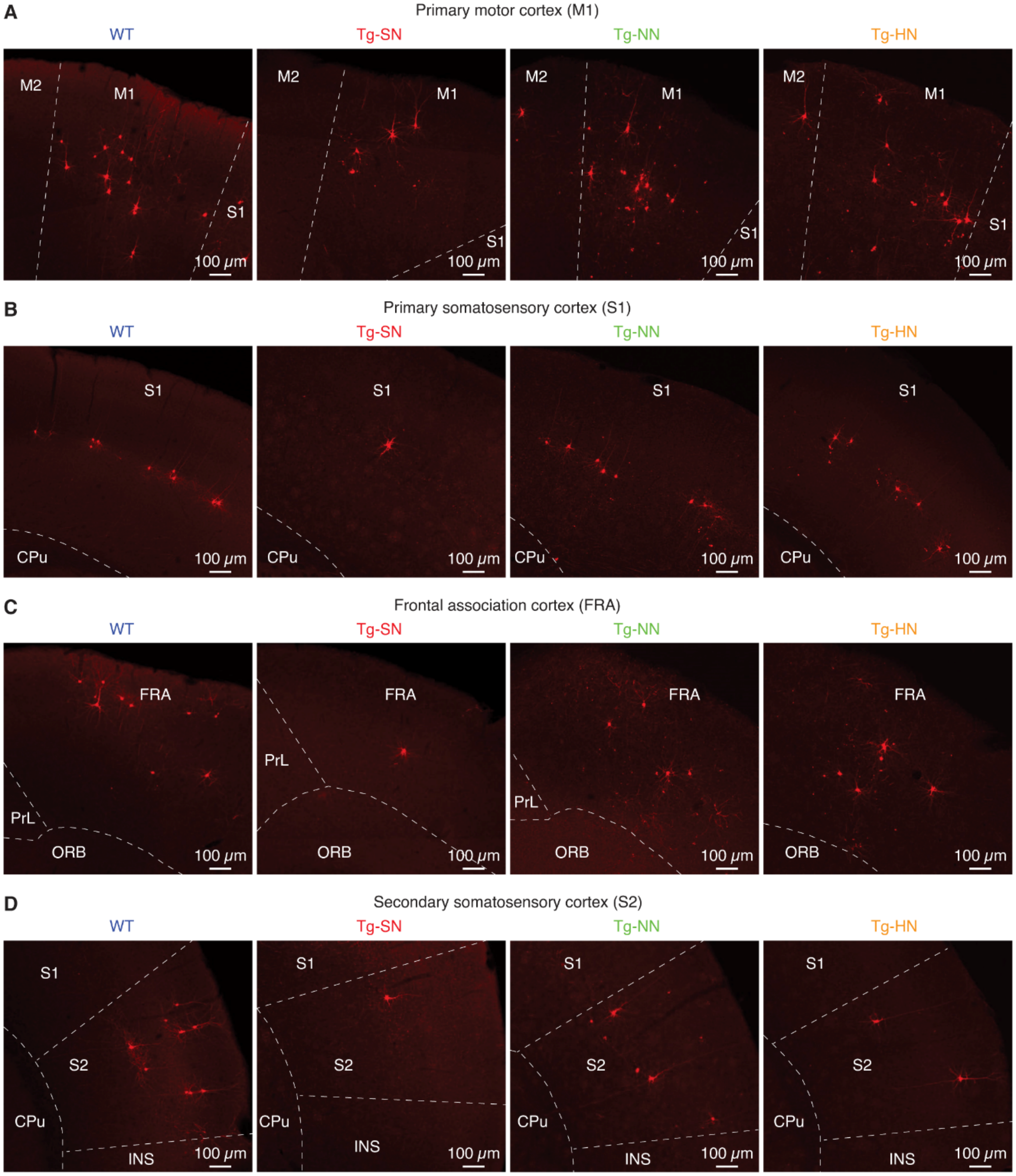

Fig.S3. Long-range monosynaptic inputs to the starter neurons. Representative confocal images of the presynaptic inputs (mCherry-expressing neurons) to a WT (far left), Tg-SN (left), Tg-NN (right), and a Tg-HN (far right) starter neuron in M2. Displayed are inputs from ipsilateral M1 (**A**), S1 (**B**), FRA (**C**), and S2 (**D**). Scale bar: 100 μm. Abbreviations: M1, primary motor cortex; M2, secondary motor cortex; S1, primary somatosensory cortex; Cpu, caudoputamen; FRA, frontal association cortex; PrL, prelimbic cortex; ORB, orbital cortex; S2, secondary somatosensory cortex; INS, insular cortex.

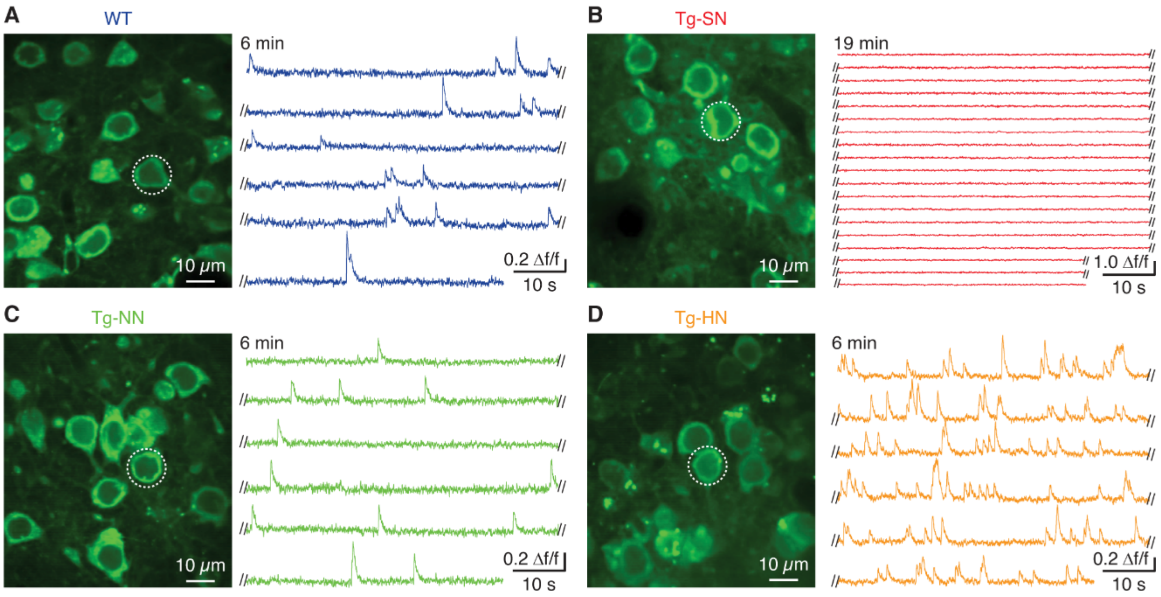

Fig.S4. Functional identification of the neurons for the experiment in Fig.3. (**A**) *Left*: In vivo two-photon image of Cal-520 AM-stained layer 2/3 neurons in M2 of a WT mouse. The circled cell corresponds to the WT neuron in Fig. 3A and E. *Right*: Spontaneous Ca^{2+} traces from 6 consecutive 1-min recordings from the neuron circled in the left panel. (**B**) *Left*: In vivo two-photon image of Cal-520 AM-stained layer 2/3 neurons in M2 of an APP23xPS45 mouse. The circled neuron in the image corresponds to the Tg-SN in Fig. 3B and F. *Right*: Spontaneous Ca^{2+} traces from 19 consecutive 1-min recordings from the Tg-SN circled in the left panel. (**C**) Same as (B) for the Tg-NN in Fig. 3C and G, 6 consecutive 1-min recordings are displayed. (**D**) Same as (C) for the Tg-HN in Fig. 3D and H. Scale bars: 10 μm

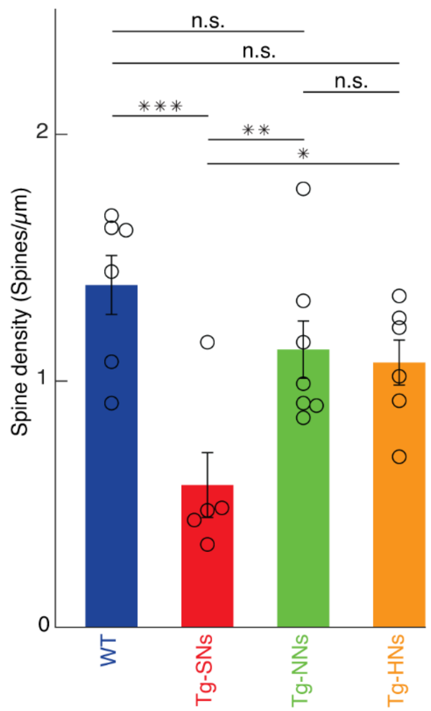

Fig.S5. Average spine density of the different neuronal types. Bar graphs depicting the average spine density of WT (*blue*), Tg-SNs (*red*), Tg-NNs (*green*), and Tg-HNs (*orange*) neurons. Each circle corresponds to an individual neuron. Error bars depict SEM. ANOVA for the effect of neuron type (WT, Tg-SN, Tg-NN, Tg-HN) on the spine density: $F(3,20) = 6.46$, $p = 0.0031$. Post-hoc tests were performed using Matlab's coeftest function. Significant differences between pairs are indicated by the p-value. *$p < 0.05$, **$p < 0.01$, and ***$p < 0.001$, n.s. not significant.

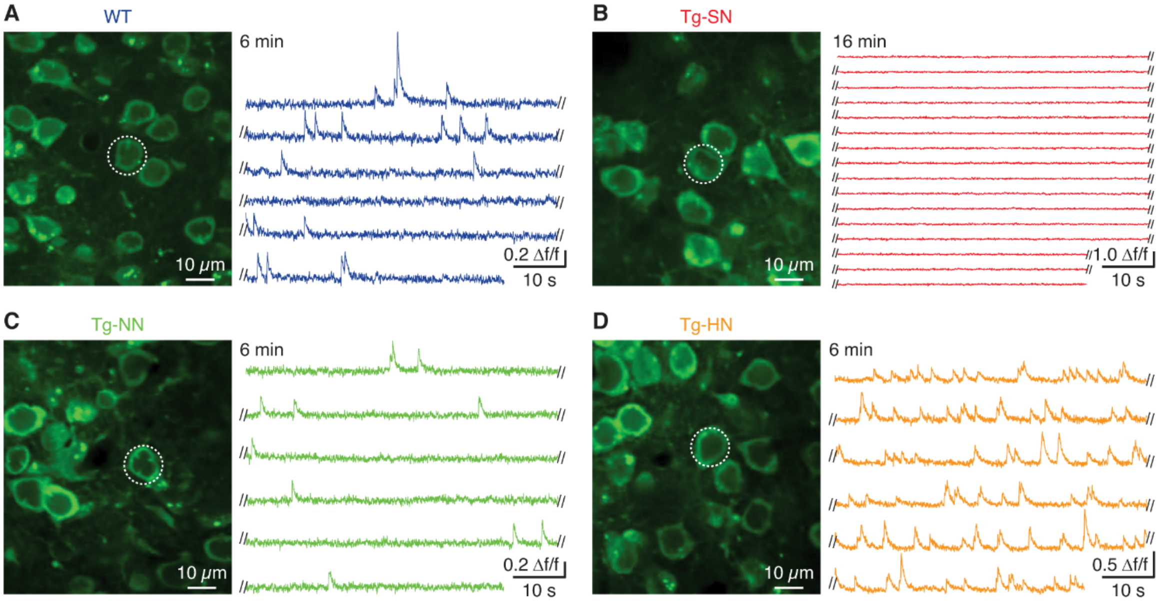

Fig.S6. Functional identification of the neurons for the experiment in Fig. 4. (**A**) *Left*: In vivo two-photon image of Cal-520 AM-stained layer 2/3 neurons in M2 of a WT mouse. The circled cell corresponds to the WT neuron in Fig. 4A. *Right*: Spontaneous Ca^{2+}-traces from 6 consecutive 1-min recordings from the neuron circled in the left panel. (**B**) *Left*: In vivo two-photon image of Cal-520 AM-stained layer 2/3 neurons in M2 of an APP23xPS45 mouse. The circled neuron in the image corresponds to the Tg-SN in Fig. 4B. *Right*: Spontaneous Ca^{2+} traces from 16 consecutive 1-min recordings from the neuron circled in the left panel. (**C**) Same as (B) for the Tg-NN in Figure 4C, 6 consecutive 1-min recordings are displayed. (**D**) Same as (C) for the Tg-HN in Figure 4D. Scale bars: 10 μm.

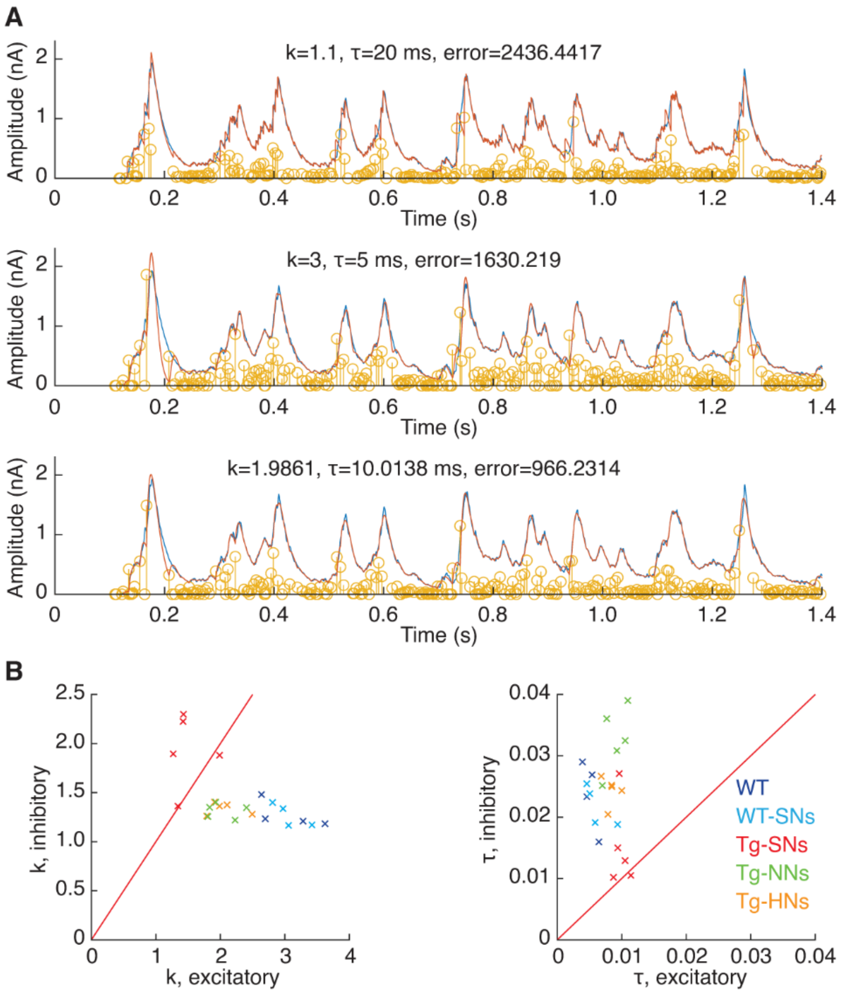

Fig.S7. Estimation of the parameters of the best-fitting unitary event for traces from whole-cell voltage-clamping recordings. (**A**) Example traces show a short current segment (*blue*) with the fit (*red*) at different parameter values acquired by a search algorithm. The top and middle cases are non-optimal fits (the top one is too coarse, and the middle one is too smooth). The bottom one is with the best parameters. The yellow stems show the location of the unitary events and their amplitudes. (**B**) Scatter plot showing the parameter k (*left*) and τ (*right*) of the best-fitting unitary events for excitatory and inhibitory currents of each neuron.

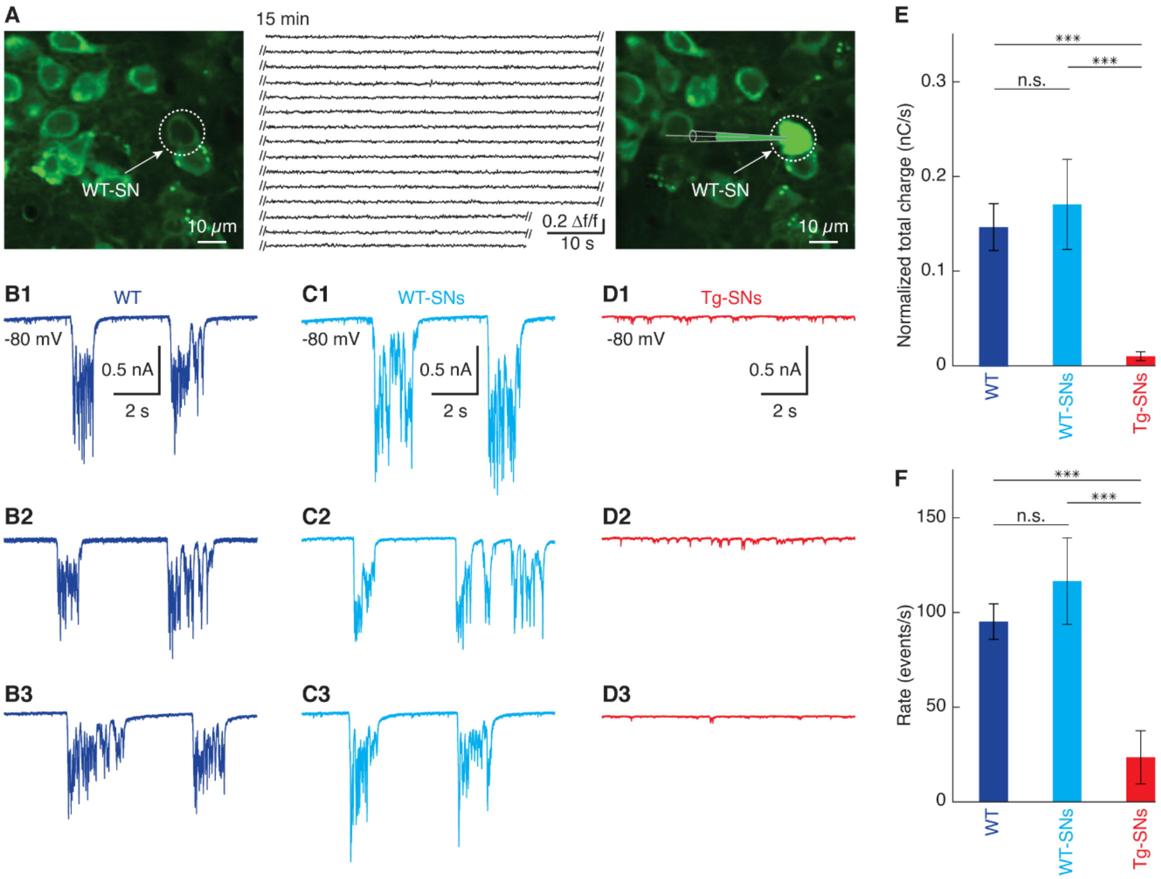

Fig. S8. Intact excitatory inputs to silent neurons in WT mice. (A) Left: In vivo two-photon image of Cal-520 AM-stained layer 2/3 neurons in M2 of a WT mouse. The circled cell corresponds to a silent neuron (WT-SN). middle: Spontaneous Ca2+ traces from 15 consecutive 1-min recordings from the neuron circled in the left panel. right: subsequent in vivo whole-cell voltage-clamp recording of the same cell. **(B)** Representative spontaneous excitatory postsynaptic currents (EPSCs) recorded in vivo from three L2/3 M2 neurons (B1-B3), from the same experimental group as the neuron in Fig. 4A. **(C)** Representative EPSCs recorded in vivo from three L2/3 M2 silent neurons in WT mice (C1-C3). **(D)** Representative EPSCs recorded in vivo from three L2/3 M2 silent neurons(D1-D3), from the same experimental group as the neuron in Fig.4D. **(E)** Bar graph showing the normalized total charge of the EPSCs obtained in neurons of the different experimental groups (for WT ($n = 4$), WT-SNs ($n = 4$), and Tg-SNs ($n = 5$). **(F)** The same as in (E) for the rate of the respective synaptic currents in the same neurons. Error bars in (E) and (F) depict SEM. Linear mixed effects model for charge/rate as a function of experimental group followed by ANOVA; effect of neuron type (WT, WT-SNs, Tg-SN) on excitatory charge (E): WT vs. TG-SN: $F(1,18) = 15.6$, $p = 0.00092$, WT-SN vs. TG-SN: $F(1,18) = 19.8$, $p = 0.00031$, WT vs. WT-SN: $F(1,18) = 0.22$, $p = 0.65$; neuron type on rate of excitatory events (F): WT vs. TG-SN: $F(1,18) = 33.2$, $p = 0.000018$, WT-SN vs. TG-SN: $F(1,18) = 18.8$, $p = 0.00040$, WT vs. WT-SN: $F(1,18) = 1.84$, $p = 0.19$. Post-hoc tests were performed using Matlab's coeftest function. Significant differences between pairs are indicated by the p-value. * $p < 0.05$, ** $p < 0.01$, and *** $p < 0.001$. n.s. not significant. Scale bars in (A): 10 μm.

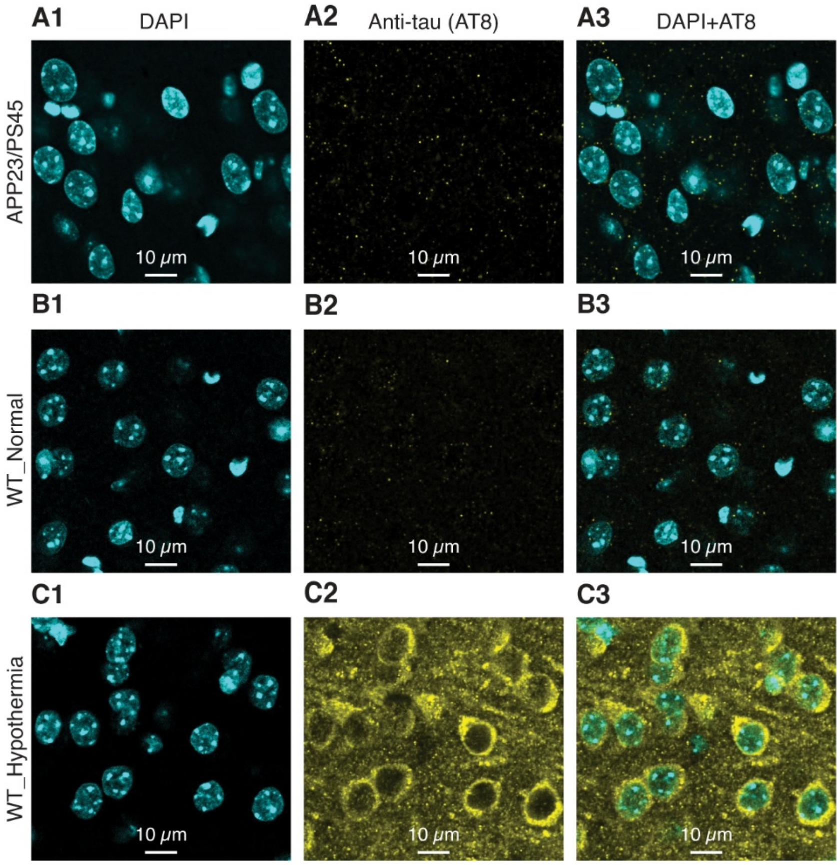

Fig. S9. Absence of hyperphosphorylated tau in neurons of APP23/PS45 mouse brain. (A) Co-staining of DAPI (cyan, A1) and the phosphorylated tau marker AT8 (yellow, A2) in M2 of an APP23/PS45 transgenic mouse. (A3) Merged image of the left and middle panels. **(B)** Co-staining of DAPI (cyan, B1) and AT8 (yellow, B2) in M2 of a WT mouse. (B3) Merged image. **(C)** Co-staining of DAPI (cyan, C1) and AT8 (yellow, C2) in M2 of a WT mouse subjected to hypothermia for 1h before perfusion. (C3) Merged image. Scale bars: 10 μm.

Cortical region / Exp. group	Ipsilateral								Contralateral							
	M2	M1	S1	FRA	S2	ORB	INS	ECT	M2	M1	S1	FRA	S2	ORB	INS	ECT
WT-1	322	266	210	228	86	27	42	6	39	39	14	35	2	3	8	0
WT-2	268	279	118	72	108	23	33	3	40	57	7	15	1	21	1	0
WT-3	254	177	127	58	41	15	9	0	33	10	3	10	0	10	0	0
WT-4	60	239	113	30	43	5	5	1	14	41	7	7	1	9	0	0
WT-5	100	59	35	72	4	6	0	0	10	1	1	1	0	0	0	0
WT-6	97	40	39	36	9	3	0	0	9	2	2	0	1	1	0	0
Tg-SN 1	13	5	4	3	0	0	0	0	0	0	0	2	0	0	0	0
Tg-SN 2	29	91	25	5	3	1	0	0	0	4	0	0	0	2	0	0
Tg-SN 3	65	48	5	6	1	1	0	0	1	2	0	1	0	0	0	0
Tg-SN 4	16	9	0	3	0	0	0	0	0	2	0	0	0	0	0	0
Tg-SN 5	13	4	1	36	1	0	0	0	0	0	0	0	0	0	0	0
Tg-NN 1	381	359	138	84	42	7	8	0	23	46	5	3	1	6	5	0
Tg-NN 2	201	297	54	12	4	5	7	0	4	7	1	1	0	8	0	0
Tg-NN 3	382	65	34	4	5	7	5	0	7	0	0	0	0	8	0	0
Tg-NN 4	195	101	42	111	7	7	4	0	10	10	0	7	0	2	0	0
Tg-NN 5	44	20	13	32	5	1	0	0	0	0	0	0	0	0	0	0
Tg-NN 6	62	13	1	18	0	0	1	0	2	0	0	0	0	0	0	0
TG-HN 1	222	358	49	42	5	5	10	0	8	17	0	2	0	2	0	0
TG-HN 2	395	83	41	20	9	0	4	0	5	4	0	0	1	3	1	0
TG-HN 3	136	219	67	16	26	15	2	0	5	6	0	5	0	4	0	0
TG-HN 4	112	42	34	106	5	1	1	0	3	10	1	3	0	0	0	0
TG-HN 5	93	49	28	74	11	6	0	0	1	5	0	1	1	6	0	0
TG-HN 6	116	33	12	17	0	0	0	0	1	5	0	0	0	0	0	0

Table S1. Region-specific distributions of neurons connected monosynaptically to single M2 neurons in the different experimental groups.
Abbreviations: M2, secondary motor cortex; M1, primary motor cortex; S1, primary somatosensory cortex; FRA, frontal association cortex; S2, secondary somatosensory cortex; ORB, orbital cortex: INS: insular cortex; ECT, ectorhinal cortex.

SI References

1. K. Franklin, G. Paxinos, *The mouse brain in stereotaxic coordinates, compact. The coronal plates and diagrams* (2004).
